# Association of immunoglobulin GM allotypes with longevity in long-living individuals from Southern Italy

**DOI:** 10.1186/s12979-018-0134-7

**Published:** 2018-11-06

**Authors:** Annibale A. Puca, Anna Ferrario, Anna Maciag, Giulia Accardi, Anna Aiello, Caterina Maria Gambino, Giuseppina Candore, Calogero Caruso, Aryan M. Namboodiri, Janardan P. Pandey

**Affiliations:** 1 Department of Medicine and Surgery, University of Salerno, Baronissi, Italy; 2 IRCCS MultiMedica, Milan, Italy; 3 Department of Pathobiology and Medical Biotechnologies, University of Palermo, Corso Tukory 211, 90134 Palermo, Italy; 4 Department of Microbiology and Immunology, Medical University of South Carolina, Charleston, SC 29425 USA

**Keywords:** GM allotypes, HMCV, HSV-1, Immune response, Longevity

## Abstract

**Background:**

The aim of this study was to analyse the role of GM allotypes, i.e. the hereditary antigenic determinants expressed on immunoglobulin polypeptide chains, in the attainment of longevity. The role played by immunoglobulin allotypes in the control of immune responses is well known as well as the role of an efficient immune response in longevity achievement. So, it is conceivable that particular GM allotypes may contribute to the generation of an efficient immune response that supports successful ageing, hence longevity.

**Methods:**

In order to show if GM allotypes play a role in the achievement of longevity, we typed the DNA of 95 Long-living individuals (LLIs) and 96 young control individuals (YCs) from South Italy for GM3/17 and GM23+/− alleles.

**Results:**

To demonstrate the role of GM allotypes in the attainment of longevity we compared genotype and allele frequencies of GM allotypes between LLIs and YCs. A global chi-square test (3 × 2) shows that the distribution of genotypes at the GM 3/17 locus is highly significantly different in LLIs from that observed in YCs (*p* < 0.0001). The 2 × 2 chi-square test shows that the carriers of the GM3 allele contribute to this highly significant difference. Accordingly, GM3 allele is significantly overrepresented in LLIs. No significant differences were instead observed regarding GM23 allele.

**Conclusion:**

These preliminary results show that GM3 allotype is significantly overrepresented in LLIs. To best of our knowledge, this is the first study performed to assess the role of GM allotypes in longevity. So, it should be necessary to verify the data in a larger sample of individuals to confirm GM role in the attainment of longevity.

## Background

The term allotype refers to any genetic variant of a protein. However, in immunology it is used for hereditary antigenic determinants expressed on immunoglobulin polypeptide chains, i.e. the genetic markers of γ chains (GM). GM allotypes are encoded by autosomal codominant alleles that follow Mendelian laws of heredity on immunoglobulin heavy chain γ1, γ2 and γ3 genes [1]. The role played by immunoglobulin allotypes in the control of immune responses was recognized 46 years ago [2]. Several studies have clearly shown that immune response to many infectious agents, vaccines, and autoantigens is associated with particular GM allotypes [3]. Moreover, the well-known differences in the frequencies of GM allotypes among different ethnic groups, and the strong linkage disequilibrium within a given ethnic group, suggest that Darwinian selection over many generations, i.e. selection by major infectious diseases, has played a role in the maintenance of polymorphisms of IGHG genes, of which some are common and others are rare [3].

On the other hand, the role of an efficient immune response in the attainment of longevity is well known [4]; hence it is reasonable to hypothesize an association of GM allotypes with longevity. Using hypothesis driven candidate gene approaches, numerous studies have identified particular GM genes as risk factors for many malignant, infectious, and autoimmune diseases, but most of these findings have not been confirmed or refuted by the genome-wide association studies (GWAS) [3]. In addition, GWAS on longevity have not demonstrated associations of these genotypes with longevity. In fact, although most GM alleles are common within an ethnic group (some with gene frequency > 70%), they are not being evaluated in the GWAS of longevity, because these determinants are not included in the commonly employed genotyping platforms. In fact, since GM allotypes were not typed in the haplotype map (HapMap) project, they cannot be imputed. Even in the 1000 Genomes project, the coverage of this region is very low, resulting in poor quality of imputation [5].

Therefore, a candidate gene approach is necessary for evaluating the possible role played by GM genes in the attainment of longevity. So, in this paper we have analysed, by classic case control study, the distribution GM allotypes in longevous people and controls from Southern Italy. To this end, we analysed the frequencies of GM3 and GM17 determinants (arginine to lysine replacement) expressed in the constant heavy (CH)1 region of IgG1 heavy chain, and GM23- and GM23+ determinants (valine to methionine replacement) in the fragment crystallisable region (Fc) of IgG2 heavy chain [1, 3].

## Results

In order to demonstrate the role of GM allotypes in the attainment of longevity and to strengthen previous results suggesting that genetic factors involved in immune responses may play a key role in longevity, we compared genotype and allele frequencies of GM allotypes between LLIs and YCs.

The genotype frequency distributions of GM3/17 genotypes and alleles are presented in Tables 1 and 2, respectively. A global chi-square test (3 × 2) shows that the distribution of the three genotypes at the GM 3/17 locus is highly significantly different in LLIs from that observed in YCs (*p* < 0.0001). The 2 × 2 chi-square test shows that the carriers of the GM3 allele contribute to this highly significant difference. Accordingly, GM3 allele is significantly overrepresented in LLIs (Table 2) (OR = 2.13; *P* = 0.0003).

**Table 1:** GM 3/17 genotypes in 95 Long-living individuals (LLIs) and Controls (YCs)

	LLI s	YCs	Chi square	P
GM 3/3	48	17	18.99	0.00001
Rest	47	70		
Total	95	87		
	LLI	Controls	Chi square	P
GM 3/17	37	57	12.83	0.0003
Rest	58	30		
Total	95	87		
	LLI	Controls	Chi square	P
GM 17/17	10	13	0.802	0.3704
Rest	85	74		
Total	95	87		

3/3 genotype OR (C.I. 2.16–8.18) = 4.21 (*P* < 0.00001)

**Table 2:** GM 3/17 Alleles in Long-living individuals (LLIs) and Controls (YCs)

	*N* = 190		*N* = 174		OR	C.I.	p
	LLIs		YCs				
	+	%	+	%			
3	133	70	91	52	2.13	1.38–3.27	=0.0003
17	57	30	83	48			

Significance of distribution by chi square test (2 × 2) *p* = 0.0005

Since it is well known that immune system ages differently in males and females [6], we analysed data separately for men and women. Results show that GM3 allele is significantly associated with longevity in both the sexes (data not shown).

The genotype frequency distributions of GM23 genotypes and alleles are presented in Tables 3 and 4, respectively. No significant differences were found for the distribution of GM23 genotypes and alleles between LLIs and YCs. Also, analysing data according to sexes, no significant differences were found in both males and females (data not shown).

**Table 3:** GM 23 genotypes in Long-living individuals (LLIs) and Controls (YCs)

	*N* = 96			*N* = 92		
	LLIs			YCs		
	+	–	%	+	–	%
23 −/−	20	76	20.8	18	74	19.6
23 +/−	38	58	39.6	39	53	42.4
23 +/+	38	58	39.6	35	57	38.0

Significance of distribution by chi square test (2 × 6) p = NS

**Table 4:** GM 23 Alleles in Long-living individuals (LLIs) and Controls (YCs)

	*N* = 192		*N* = 184	
	LLIs		Controls	
	+	%	+	%
23 +	114	59.4	109	59.2
23 -	78	40.6	75	40.8

Significance of distribution by chi square test (2 × 2) p = N.S

## Discussion

The LLIs, i.e. those approaching 100 years of age, are a model of successful ageing. The exceptional longevity of LLIs is to some extent genetically guided, as emphasized by the family clustering of extreme longevity and the reduced mortality of the centenarian siblings compared to age-related elderly [7]. Longevity genes can be discovered by genetic association studies or GWAS conducted on LLIs [7, 8]. These kinds of studies identify the gene variants that have been selected by the demographic pressure and, therefore, are somehow significant for human health. The identification of these genetic variants is important since they could represent potential targets for fighting ageing-related diseases. Several research groups are working on genes involved in oxidative stress, in lipid and glucose metabolism, in immune-inflammatory responses, in DNA damage and in repair, in nutrient sensing pathways. Many results have been obtained in association studies of candidate genes, but other results are still in conflict. In particular, to date, the majority of GWAS, that rely on large population sets for multiple testing and power issues, have only confirmed the decreased frequency of detrimental alleles of apolipoprotein E (APOE) and the increase of protective alleles of forkhead box O3A (FOXO3A) with some exceptions [7–10].

In the present paper we have analysed, by a classic case control study, the distribution of GM allotypes, in longevous people and controls from Southern Italy. Data show that GM3 allotype is significantly overrepresented in LLIs. To the best of our knowledge, no study has evaluated GM allotype role in human longevity. Since the distribution of GM allotypes in the population under study is not known, a note of caution must be taken into account, because of the relatively small sample sizes of LLIs and controls. However, a study performed on the sheep several years ago reported an influence of IgG3 allotypes in ageing of sheep. These findings, showing the role played by GM allotypes in other animal species, fit ours [11].

A study by de Vries et al. [12] is relevant to our findings. Descendants of Dutch colonists, who emigrated to Surinam and survived epidemics of typhoid and yellow fever with a total mortality of about 60%, were tested for different polymorphisms, including GM allotypes, whose frequencies were compared with those of Dutch control sample. Several polymorphisms, including GM allotypes, were shown to influence the chance for survival, indicating selection through genetic control of immune response to pathogens. Another example of such selection is the longevity associated variant of Bactericidal/Permeability-increasing (BPI) Family B member 4 (BPIFB4), which belong to a family of proteins, BPI and lipid transfer protein (LTP), involved in the activation of toll-like receptor(TLR)-4 in the innate immune response [13].

So, it is conceivable that particular GM allotypes may contribute to the generation of a dynamic immune response that supports successful ageing, hence longevity, whereas certain other allotype combinations may produce suboptimal levels of immunity and reduced life span.

In particular, GM allotypes have been shown to be involved in the immunological control of viruses, including herpes simplex virus(HSV)-1 and human cytomegalovirus (HCMV) [14, 15]. Herpes viruses, such as HCMV and HSV-1, have been associated with a variety of health problems, including cognitive decline, and overall mortality in the elderly [16–19]. Accordingly, recent data show that effective control of herpes viruses is impaired during healthy ageing, most probably due to loss of cellular control of early viral reactivation [20–22].

Recently, an interplay between particular GM and cluster of designation(CD)16A alleles in the outcome of HSV-1 infection has been demonstrated. The CD16A-158 *V*/V genotype associates with an asymptomatic course of HSV-1 infection only in homozygotes for the GM3 allele. Additional studies to determine the mechanism underlying this association showed that CD16A-158 V and GM3 alleles epistatically enhanced the antibody-dependent cell-mediated cytotoxicity (ADCC) against opsonized HSV-1-infected fibroblasts [14].

Concerning HCMV, in different populations it has been demonstrated the association of human IgG1 allotypes with immune response to the virus and some kinds of cancer thought to be associated with HCMV [23, 24]. More interestingly, in another Southern Italy population, GM17/17 (the alternative allele of GM3) was associated with the risk of developing HCMV symptomatic infection [15]. It is well known the role of chronic infections from herpes viruses, in particular from HCMV, in the impairment of immune responses of elderly people, hence contributing to immunosenescence [25, 26]. Accordingly, the severity of many infections is higher in the elderly compared to younger adults and infectious diseases are frequently associated with frailty and death. Reciprocally, an immune good function is tightly correlated to health status and longevity [4, 27–29].

On the other hand, our immune system is known to be quite efficient in fighting acute infections in young people, but not particularly efficient in responding to chronic stimuli, especially when they occur late in life. This leads to an increased production of inflammatory mediators. So, this chronic antigenic stress contributes to determine an inflammatory status called inflamm-ageing, responsible for age-related diseases. Reciprocally, the control of inflamm-ageing is associated with longevity [4, 30–33]. Accordingly, an efficient control of Herpes Virus chronic infections by GM allotypes might contribute to observed association with longevity through a decrease of inflammatory status.

Several mechanisms have been proposed to explain the role played by GM allotypes in the control of virus infections. IgG allotypes might modulate avidity of the interaction of IgG with Fcγ receptor (FcγR), so influencing the efficacy of immune response. In addition, they might modulate the strength of ADCC, thus involving cells of innate response such as natural killer (NK) cells [2, 3].

## Conclusion

This is the first report implicating GM allotypes in longevity. It needs to be replicated in an independent and larger multi-ethnic study population. However, it has been reported that the association with longevity of other genes related to control of immune response as human leukocyte antigens (HLA) and killer immunoglobulin-like receptors (KIR), is population specific, being heavily affected by the population-specific genetic and environmental history [34]. On the other hand, it is also possible that there are other life span-associated loci on chromosome 14q32 (where the GM genes are located), distinct from GM, whose alleles are in significant linkage disequilibrium with those of the GM loci. This putative linkage disequilibrium could give rise to the associations observe.

## Methods

We genotyped 96 Long-living individuals (LLIs) (40 female, mean age 96.7, age-range 91–104; 56 male, mean age 93.6, age range 90–104 years) and 96 young control individuals (YCs) (66 female, mean age 31.9, age-range 20–44; and 30 male, mean age 35.2, age range 23–45 years) already recruited as part of the Southern Italian Centenarian Study [35]. The LLIs were thoroughly investigated for demographic characteristics, medical history (past and present diseases), level of independence and cognitive status. All subjects donated blood samples for DNA study and gave written informed consent to the study, which was approved by Ethical Committee of Salerno University. All methods were performed in accordance with the relevant guidelines and regulations. The study was conducted in accordance with the ethical principles that have their origins in the Declaration of Helsinki.

DNA was obtained from peripheral blood leukocytes by the salting-out technique and stored until the use. For the determination of GM3 and GM17 allotypes (A to G substitution), a direct DNA sequencing method, Sanger sequencing, was used. The DNA segment encoding the CH1 region of γ1 chain was amplified by polymerase chain reaction (PCR) according to Balbìn et al., [36] using the following primers: 5’ CCCCTGGCACCCTCCTCCAA 3′ and 5’ GCCCTGGACTGGGGCTGCAT 3′. The purified double-stranded PCR product (364 bp) was subjected to automated DNA sequencing on an ABI PRISM 3730xl DNA Analyzer. IgG2 markers GM 23− and 23+ (a G to A substitution in the Fc of the γ2 chain) were genotyped by a nested PCR-restriction fragment length polymorphism(RFLP) method [37]. IgG3 markers GM5 and GM21 were not typed, because TaqMan genotyping assays for the IgG3 allotypes are not yet available. Due to technical problems, it was not possible to type for GM3/17 one LLI and nine YCs and fror GM23 four YCs. Because of almost absolute linkage disequilibrium at GM loci within an ethnic group, subjects positive for the IgG1 allotypes GM3 and GM17 are most likely positive for the IgG3 allotypes GM5 and GM21 (Pandey, unpublished observations).

Allele and genotype frequencies among groups were estimated by gene counting. The different chi-squares tests were used to detect significant changes in genetic variables between groups where appropriate. Standard odds ratios (OR) with 95% confidence interval (CI) were calculated.

## Abbreviations

ADCC: Antibody-dependent cell-mediated cytotoxicity
APOE: Apolipoprotein E
BPIFB4: Bactericidal/Permeability-increasing Family B member 4
CD: Cluster of designation
CH: Constant heavy
CI: Confidence interval
Fc: Fragment crystallisable region
FcγR: Fcγ receptor
FOXO3A: Forkhead box O3A
GM: Genetic markers of γ chains
GWAS: Genome-wide association studies
HapMap: Haplotype map
HCMV: Human cytomegalovirus
HLA: Human leukocyte antigen
HSV: Herpes simplex virus
KIR: Killer immunoglobulin-like receptor
LLI: Long-living individual
LTP: Lipid transfer protein
NK: Natural killer
OR: Odd ratios
PCR: Polymerase chain reaction
RFLP: Restriction fragment length polymorphism
TLR: Toll-like receptor
Yc: Young control individual
